# Charcot-Marie-Tooth Disease Presenting in the Postpartum Period: A Case Report

**DOI:** 10.7759/cureus.76077

**Published:** 2024-12-20

**Authors:** Husameldein Ismail, Bawani Anand, Tom Hughes, Benny Thomas

**Affiliations:** 1 Clinical Neurophysiology, University Hospital of Wales, Cardiff, GBR; 2 Neurology, University Hospital of Wales, Cardiff, GBR

**Keywords:** caesarean, charcot-marie-tooth, genetic testing, inflammatory neuropathy, nerve conduction study (ncs), neuropathy in pregnancy, pmp22 gene, postpartum

## Abstract

Charcot-Marie-Tooth disease (CMT) is the most common hereditary peripheral neuropathy. It presents a wide range of genetic and phenotypic heterogeneity. CMT disease type 1A (CMT1A), caused by PMP22 gene duplication, represents the most common subtype of CMT in Western countries. It usually presents insidiously with stigmata of chronic neuropathy. We report a rare case of CMT1A, which presents acutely in the postpartum period. A young woman who experienced acute bilateral lower limb numbness and weakness post-caesarean section, which was initially attributed to spinal anaesthesia. The initial work-up was normal, but subsequent nerve conduction studies indicated a possibility of hereditary, demyelinating sensory-motor neuropathy. Genetic testing confirmed the diagnosis of CMT1A. The article explores the effects of pregnancy on patients with CMT in terms of potential worsening or unmasking of CMT symptoms and the development of obstetric complications in these patients. It also sheds some light on the role of electrophysiological studies and various biomarkers to differentiate between acquired and hereditary neuropathy. In conclusion, it emphasizes the importance of considering hereditary neuropathies in the differential diagnosis of acute atypical neurological presentation during the postpartum period.

## Introduction

Charcot-Marie-Tooth disease (CMT, also referred to as hereditary motor and sensory neuropathy) encompasses a group of hereditary peripheral neuropathies characterized by slowly progressive motor and sensory nerve dysfunction. It is named after the three neurologists who first described the condition in 1886. It is the most common hereditary peripheral neuropathy, with a variable prevalence among countries, ranging from 9.7/100,000 to 82.3/100,000 [1,2]. CMT is caused by various pathogenic gene variants expressing protein products in myelin, gap junctions, and/or axonal structures within the peripheral nerves.

CMT is classified into several types, including CMT1 through CMT4 and an X-linked category (CMTX), with further subtypes labelled with numbers and letters (A, B, C, ...). These types are classified based mainly on their clinical features, modes of inheritance, and neurophysiological findings. It is closely related to two other, less common inherited neuropathies: distal hereditary motor neuropathies (HMN), which affect only motor nerves, and hereditary sensory neuropathies (HSN), which primarily involve sensory nerves. These three disorders, CMT, HMN, and HSN, are often collectively referred to as CMT [3].

The mode of inheritance in CMT varies from autosomal dominant to autosomal recessive to X-linked. Over 60 pathogenic variants have been associated with CMT [3], including whole-gene duplications, deletions, and single-nucleotide variants. This genetic diversity further complicates diagnosing CMT, as it includes a broad, heterogeneous group of genetic neuropathies with significant variation and occasional lack of genotype-phenotype correlation. Patients exhibit intra-familial and inter-familial phenotypic heterogeneity, even among families with identical genetic abnormalities [4].

Duplication of the PMP22 gene on chromosome 17p.11 causes CMT1A, the most common subtype in Western countries, accounting for around 50% of all CMTs [5]. Point mutations of this gene have also been described as a cause of CMT1A, although not very frequent (2-5% of CMT1A cases) [6]. Age at onset ranges from infancy to the elderly, but most patients have onset within the first two decades of life. Symptoms include unsteady gait, pes cavus, muscle cramps, foot drop, retraction of the Achilles tendon, weakness and atrophy of the intrinsic foot and peroneal muscles, “high-stepping” gait, claw hand in extreme cases, generalized areflexia (early feature), scoliosis (10%), and nerve hypertrophy (25-66%). The neuropathy in CMT is typically slowly progressive, with symptoms starting in the distal extremities and spreading proximally over time(length-dependent).

We present a rare and unusual case of CMT1A that presented acutely during the postpartum period. This case provides valuable lessons for clinical practice. 

## Case presentation

The patient is a young woman in her third decade of life with no significant past medical or family history. She is not taking any regular medications. Her first pregnancy, four years earlier, was neurologically uneventful and without complications.

She delivered her second baby by uneventful caesarean section under spinal anaesthesia. In the following days, she experienced numbness and weakness in her legs. Initially, her symptoms were attributed to the effect of spinal anaesthesia. An inpatient workup, including full blood count, urea and electrolytes, liver function tests, thyroid function tests, bone profile, vitamin D, vitamin B12, folate, and a lumbosacral MRI, revealed no abnormalities. It is worth noting that a lumbar puncture was not performed at that time. She was discharged home from the maternity ward. At home, she struggled with mobility and developed pressure sores on her buttocks. After about four weeks, she started spontaneously recovering and could walk without assistance in four months.

Four months after her second delivery, she attended the neurophysiology clinic, walking independently with no neurological deficits apart from bilateral impaired pinprick sensation to her mid-calves. There were no stigmata of chronic neuropathy in her legs. The first nerve conduction study (NCS) revealed a demyelinating sensory-motor neuropathy with partial conduction blocks in two nerves, affecting the lower limbs more than the upper limbs (see Tables 1-2). Another two follow-up nerve conduction studies, conducted around six and 14 months later, revealed persistent demyelinating changes but with a resolution of the partial conduction blocks noted in the upper limbs and improved motor amplitudes. They were consistent with a uniform, demyelinating sensory-motor neuropathy (see Tables 3-4). Then, targeted gene testing using multiplex ligation-dependent probe amplification (MLPA) was decided upon, which demonstrated a heterozygous pathological duplication of the PMP22 gene, confirming a diagnosis of CMT disease type 1A (CMT1A).

**Table 1:** First nerve conduction study. Sensory conduction studies in the upper and lower limbs. It shows a profoundly slowed conduction velocity, prolonged latencies, and moderately reduced sensory amplitudes of the median and ulnar nerves. Radial, superficial fibular, and sural sensory responses are absent, apart from the severely reduced sensory amplitude of the right radial nerve. Abbreviations: CV, conduction velocity; NR, non-recordable

Left	Latency (ms)	Amplitude (μV)	Distance (cm)	CV (m/s)
Median	4.4	3	12	27
Ulnar	3.7	4	9	24
Radial	NR	NR	-	NR
Superficial fibular	NR	NR	-	NR
Sural	NR	NR	-	NR
Right	-	-	-	-
Median	4.6	4	12	26
Ulnar	3.5	5	9	29
Radial	NR	NR	-	NR
Superficial fibular	NR	NR	-	NR
Sural	NR	NR	-	NR

**Table 2:** First nerve conduction study. Motor conduction studies in the upper and lower limbs. It shows prolonged distal latencies, delayed to absent F-latencies, partial conduction blocks of the median nerves, slowed conduction velocity of the median and ulnar nerves, and preserved CMAP amplitudes of the ulnar nerves. Fibular and tibial nerves CMAPs are absent/ severely reduced. Abbreviations: CV, conduction velocity; NR, non-recordable; EDB, extensor digitorum brevis; AHB, abductor hallucis brevis; CMAP, compound muscle action potential

Left	Latency (ms)	Amplitude (mV)	Distance (cm)	CV (m/s)	F latency
Median/wrist	6.6	2.8	-	-	39.4
Median/elbow	12.2	0.86	21.5	33	NR
Ulnar/wrist	4.4	6.1	-	-	37.7
Ulnar/elbow	11.5	7.2	22	31	-
Fibular (EDB)	NR	NR	-	NR	NR
Tibial (AHB)	7.6	0.15	-	-	NR
Right	-	-	-	-	-
Median/wrist	4.7	12.6	-	-	38.9
Median/elbow	11.6	7.0	20	29	-
Ulnar/wrist	4.3	5.3	-	-	NR
Ulnar/elbow	11.9	5.0	26	34	-
Fibular (EDB)	6.9	0.12	-	-	NR
Tibial (AHB)	10.6	0.31	-	-	NR

**Table 3:** Third nerve conduction study. Sensory conduction studies in the upper and lower limbs. Median and ulnar nerves show markedly slowed conduction velocity, prolonged latencies, and mildly reduced sensory amplitudes. Sural sensory responses are absent. Abbreviations: CV, conduction velocity; NR, non-recordable

Left	Latency (ms)	Amplitude (μV)	Distance (cm)	CV (m/s)
Median	4.1	6	12.5	30
Ulnar	3.2	8	8.5	27
Sural	NR	NR	-	NR
Right	-	-	-	-
Median	3.8	8	11.5	30
Ulnar	3.2	6	9.5	27
Sural	NR	NR	-	NR

**Table 4:** Third nerve conduction study. Motor conduction studies in the upper and lower limbs. It shows prolonged distal latencies, prolonged F-latencies, preserved CMAP amplitudes, and slowed conduction velocity of all the tested nerves. Note the resolution of the previous partial conduction blocks of the median nerves. Abbreviations: CV, conduction velocity; EDB, extensor digitorum brevis; AHB, abductor hallucis brevis; POP, popliteal fossa

Left	Latency (ms)	Amplitude (mV)	Distance (cm)	CV (m/s)	F latency
Median/wrist	6.4	7.5	-	-	39.2
Median/elbow	12.9	7.5	20.5	32	NR
Ulnar/wrist	3.3	8.7	-	-	39.6
Ulnar/elbow	11.9	6.1	25.0	29	-
Fibular (EDB)/ankle	7.2	3.1	-	-	87.3
Fibular (EDB)/POP	20.5	1.8	38.5	29	-
Tibial (AHB)	8.2	11.3	-	-	60.8
Right	-	-	-	-	-
Median/wrist	5.7	13.8	-	-	39.4
Median/elbow	12.5	12.4	20.5	30	-
Ulnar/wrist	4.5	9.9	-	-	37.7
Ulnar/elbow	12.6	8.3	28	35	-
Fibular (EDB)/ankle	7.0	1.92	-	-	NR
Fibular (EDB)/POP	22.5	1.49	38	25	-
Tibial (AHB)	13.2	5.8	-	-	77.7

## Discussion

Several studies have reported worsening of CMT symptoms during pregnancy, with incidence ranging from 16.3% to 38% [7,8]. The data on obstetric complications in CMT patients are variable in the literature. Some studies indicated a higher risk of abnormal presentations, postpartum hemorrhage, and operative deliveries. For example, a meta-analysis study conducted at the National Hospital for Neurology and Neurosurgery in London found that 37% of pregnant patients with CMT experienced worsening CMT symptoms, with half of them improving after delivery [6]. Two other large retrospective studies using self-reported questionnaires were conducted in Italy and Germany [7,8]. The Italian study concluded higher rates of placenta previa, abnormal presentations, and preterm deliveries compared to the reference population. By contrast, the German study found no significant differences in obstetric complications compared to the reference population.

Occasionally, it might be challenging to differentiate between CMT and an acquired inflammatory neuropathy. Epidemiological data from some studies reveals that CMTs are more frequent than inflammatory or paraneoplastic neuropathies. An acute or subacute onset of patchy neuropathy with conduction block and temporal dispersion typically suggests an acquired demyelinating neuropathy, such as chronic inflammatory demyelinating polyradiculoneuropathy (CIDP) or Guillain-Barré syndrome. However, certain genetic types of CMT (GJB1, MPZ, SH3TC2, SPTLC1, and FIG4 mutations) can present with neurophysiological features very similar to an acquired inflammatory neuropathy. This is most commonly encountered in CMTX1 due to GJB1 mutations and should be considered in patients with treatment-resistant CIDP [9,10]. Traditionally, median nerve conduction velocity (NCV) has been utilized as a sensitive test to differentiate demyelinating from axonal CMT. NCV <38 m/s indicates a demyelinating CMT, while NCV>38 m/s suggests an axonal variant. There is also a third category, intermediate CMT, where the conduction velocities are between 25 and 45 m/s. Reduction in the amplitudes of motor evoked potentials (MEP) and somatosensory evoked potentials (SSEP)reflects the degree of axonal loss, which correlates with the clinical disability even in subjects with demyelinating CMTs. Also, NCV can help to assess the severity of demyelination in CMTs by categorizing it according to NCV into (1) very slow (<15 m/s), (2) slow (15 to 35 m/s), (3) intermediate (35 to 45 m/s), and (4) normal (>45 m/s). Nerve conduction study is also useful to investigate axonal variants of CMT as it can categorize them as motor predominant (hereditary motor neuropathy (HMN)), sensory predominant (hereditary sensory neuropathy (HSN)), and mixed sensory-motor (CMT2). Conduction blocks and temporal dispersion phenomena are rare in CMT1A. Secondary axonal degeneration can be a co-existing feature in neurophysiological studies. In addition, several clinical markers can assist in differentiating genetic from acquired neuropathies, including asymmetric weakness, high CSF protein, and the presence of thickened nerve roots on MRI. However, these markers are not exclusive to acquired inflammatory neuropathies. Up to 7% of patients with CMT1A may exhibit minor asymmetry, and a CSF protein level of up to 1 g/L may occur. Moreover, thickened nerve roots can be seen in demyelinating neuropathies of CMT1A.

This case of CMT1A presents several unusual features, making it rare in the literature on hereditary peripheral neuropathies. The initial acute presentation in the postpartum period is particularly unusual, as CMT1A typically presents insidiously and progressively with musculoskeletal manifestations such as calf muscle atrophy and pes cavus. In this case, the peripartum period appeared to unmask latent disease. In addition, the rapid development of pressure sores (in the sacral region) in the postpartum period is atypical and indicates significant underlying neuropathy. The patient's clinical improvement and conduction blocks in the initial nerve conduction study suggest a potentially reversible inflammatory process superimposed on the final diagnosis of CMT1A. Subsequent nerve conduction studies revealed a persistent, uniformly demyelinating sensory-motor neuropathy, highlighting the need for genetic testing which confirmed CMT1A. Concomitant illnesses (not found in this patient) such as hypothyroidism, diabetes, obesity, and toxins exposure are other factors mentioned in the literature that can cause clinical deterioration in CMT patients. The negative effects of progesterone have been documented in the literature. For example, a clinical trial conducted on rats concluded that progesterone can cause overexpression of PMP22, leading to increased Schwann cell pathology and exacerbation of the clinical phenotype of CMT1A [11].

## Conclusions

These observations emphasize the importance of considering hereditary neuropathies such as CMT1A in patients who present with atypical neurological symptoms during the postpartum period. Such patients require a thorough evaluation, including genetic testing. In addition, genetic testing for appropriate CMT mutations should be considered in treatment-resistant inflammatory demyelinating neuropathies. The article also underscores the importance of serial nerve conduction studies in differentiating between acquired and hereditary neuropathies, which can uncover a latent, asymptomatic hereditary neuropathy. Interestingly, this case raises the question of the extent of genetic predisposition underlying the existing cases of acquired inflammatory demyelinating polyneuropathies. Significant gaps exist in the epidemiological studies of CMT globally. This may be related to variations in the quality and methodologies of existing studies, making it difficult to reach definitive conclusions. Therefore, additional research is necessary to explore the epidemiological features of CMT around the globe.
